# The Role of School Health Promotion in Students’ Dietary Intake during School Hours: A Qualitative Comparative Analysis

**DOI:** 10.3390/nu16131981

**Published:** 2024-06-21

**Authors:** Lisanne Vonk, Pepijn van Empelen, Tim Huijts, Iris Eekhout, Maria Jansen

**Affiliations:** 1 Department of Health Services Research, Care and Public Health Research Institute (CAPHRI), Maastricht University, P.O. Box 616, 6200 MD Maastricht, The Netherlands; maria.jansen@maastrichtuniversity.nl; 2 Academic Collaborative Center for Public Health Limburg, Public Health Service South Limburg, P.O. Box 33, 6400 AA Heerlen, The Netherlands; 3 Expertise Center Child Health, Netherlands Organisation for Applied Scientific Research (TNO), P.O. Box 3005, 2301 DA Leiden, The Netherlands; pepijn.vanempelen@tno.nl (P.v.E.); iris.eekhout@tno.nl (I.E.); 4 Research Centre for Education and the Labour Market (ROA), School of Business and Economics, Maastricht University, P.O. Box 616, 6200 MD Maastricht, The Netherlands; t.huijts@maastrichtuniversity.nl

**Keywords:** qualitative comparative analysis, dietary intake, schools, health promotion

## Abstract

Many children in the Netherlands do not adhere to dietary guidelines. Therefore, the Healthy School (HS) program stimulates healthier dietary intake of students through schools. However, evaluating the effectiveness of school health promotion in improving dietary intake is challenging due to the influence of contextual factors. Qualitative Comparative Analysis (QCA) considers these contextual factors. Therefore, we performed a QCA to examine which (combinations of) contextual factors contribute to the healthier dietary intake of students during school hours in primary schools (approximate age range children 4–12 years) and secondary schools (age range 12–18 years) when implementing the HS program for nutrition. Data were collected mainly through interviewing school staff and a school-level questionnaire in fifteen primary schools and twelve secondary schools. We included five factors for primary schools: implementation of the HS program for nutrition, degree of implementation, socioeconomic status, parental support, and student support. For secondary schools, we included school environment instead of parental and student support. For primary schools, the best results were obtained if the HS program for nutrition was implemented in high socioeconomic status schools with a combination of high implementation, parental support, and student support. Findings indicate that if secondary schools have an impeding environment and low socioeconomic status, implementation of the HS program for nutrition can result in healthier dietary intake.

## 1. Introduction

Overweightness among children and adolescents continues to be a global public health issue [1]. Overweight children are more likely to have poorer mental health [2] and are also more prone to develop chronic diseases at a later age [3]. An important contributing factor to becoming overweight is having unhealthy dietary habits [4]. Therefore, in order to foster healthier lifestyles among students, the World Health Organization (WHO) created the Health Promoting Schools framework [5], which adopts a whole-school approach, since children and adolescents spend a substantial amount of their time at school. A whole-school approach focused on nutrition is also needed in the Netherlands, since a study of RIVM showed that many children and adolescents do not adhere to the national dietary guidelines [6]. The findings of this study also showed that children consumed sugar, confectionery, and savory snacks more than adults. Therefore, school health promotion (SHP) is necessary as this could promote a healthier dietary intake of students [7].

Nevertheless, evaluating the effectiveness of SHP is complex, especially whole-school approaches such as the Health Promoting Schools framework on dietary intake. This framework does not consist of a single intervention but focuses on a combination of different intervention components: environment, school policy, health education, and skills, involving the community, including parents, and access to health services [5]. Not only are health-promoting programs complex, but so too is the setting in which they are implemented. Schools are an example of complex adaptive systems [8,9] and consist of components that are continuously interacting. Hence, implementing SHP can, therefore, have varying effects in different school contexts. Besides within-school factors, contextual school factors outside the school environment should be taken into account. The implementation of SHP can be influenced by many different contextual factors [10], and incorrect or superficial implementation could cause negative results, while positive effects may only occur if the intervention is implemented fully and as intended [11]. For example, the socioeconomic status (SES) of families may moderate the impact of SHP on dietary intake in students because of their living environment [12], and lower SES households may have more monetary constraints to purchase fruit or vegetables [13,14], or less knowledge about nutrition [15,16]. Besides the social environment, the physical environment can also be of influence since the presence of food suppliers such as supermarkets near schools might affect the impact of SHP [17].

To capture the complex nature of the school context more accurately and take into account these factors, a complex systems perspective has been proposed, although there is no consensus on how this complexity can be studied optimally [18]. To study the impact of public health measures, a relatively newer method in this field is gaining popularity, namely, Qualitative Comparative Analysis (QCA) [19]. QCA is considered a useful method to examine complex causality and provide insight into underlying (interacting) mechanisms. This analysis method provides the opportunity to combine qualitative and quantitative data, and an important strength of this analysis method is that it can take into account contextual factors [19].

Therefore, to contribute to the existing literature on the role of the school context in SHP, we evaluated the Healthy School (HS) program in the Netherlands [20], which overlaps to a great extent with the Health Promoting Schools framework of the WHO, by performing a QCA. The HS program focuses on different health topics, one of them being nutrition. Specifically for nutrition, the HS program enables schools to encourage healthier dietary habits among students of primary, secondary, and secondary vocational schools. If a school fulfills the advised requirements for nutrition for four pillars, i.e., the environment (such as an HS canteen), health education, signalizing health problems (e.g., regular monitoring of students’ dietary intake and weight status), and (dietary) school policy, a school can apply for the nutrition certificate of the HS program. If granted, schools receive the general HS program certificate as well. For this study, we answered the following research question: Which (combinations of) contextual factors contribute to the healthier dietary intake of students during school hours in primary and secondary schools when implementing the HS program for nutrition?

## 2. Materials and Methods

### 2.1. Study Design and Study Population

For this study, primary and secondary schools were recruited from eight regional Public Health Services in the Netherlands. To obtain variety in our outcome, we selected schools based on an SES indicator as a proxy for dietary intake, since these data are publicly available and because socioeconomic status was identified in our previous studies as an important determinant for lifestyle and health, as well as in previous literature [21,22]. We used different recruitment strategies, but the majority of schools were recruited by the HS adviser from the regional Public Health Service, who often was already in contact with the school. Special primary education schools and schools for practical education were also included.

### 2.2. Measurements

#### 2.2.1. Data Collection

Recruitment started in January 2022 and data collection took place between February and December 2022. We recruited sixteen primary schools (approximate age range children 4–12 years) and fifteen secondary schools (age range 12–18 years). The current study was part of a larger evaluation study of the Dutch HS program [23], for which a questionnaire was developed to measure the degree of implementation of whole-school health promotion [24]. This questionnaire was usually filled out a few days prior to a school visit by one of the school employees who was most familiar with the school’s approach regarding health promotion. During the school visit, we conducted interviews with one to four school employees, which generally took around sixty to ninety minutes. These school employees were teachers, principals or board members, guidance counselors (primary school), healthcare coordinators (secondary school), or others. We developed a semi-structured interview guide with open-ended questions that focused on the dietary intake of students during school hours, the degree of implementation of SHP, the school context (in and outside the school), and other conditions that stimulated or obstructed achieving improved student health. Questions referred to the twelve months prior to the interview, but the focus was not on COVID-19 or its impact. To collect dietary information on the students, we asked an open-ended question to the school staff about what students usually consumed during school hours. Based on their answer, follow-up questions were asked. Directly before or after visiting the school, we explored the area around the school focusing on the physical environment, such as the distance to supermarkets. During these observations, we took pictures of the school and its surroundings, such as the food supply in the school canteen. We also collected documents, such as the school guide, and in most cases, we arranged a phone call with the HS adviser of the regional Public Health Service, who was often in contact with the school to motivate and support schools by implementing the HS program. Additionally, the HS organization provided data regarding HS certification. After data collection, a coding tree was developed for data extraction. The first transcript was coded in Nvivo 12 Pro by three researchers individually and discussed afterward to discuss potential ambiguities in data coding.

#### 2.2.2. Outcomes

We focused on the dietary intake of students during school hours on a regular school day. In each primary school, we evaluated six categories of dietary intake: (1) fruit, (2) bread or snacks, (3) cookies, (4) water, (5) sugar-sweetened beverages, and (6) treats for birthdays. It can differ between schools whether students have lunch at school or at home. If students have lunch at school in the Netherlands, they usually bring their own food from home, usually bread. It is uncommon to have a warm meal during lunch. A school could obtain a ‘+’, ‘+−’, or ‘−’ for every category, or a missing value in case no or too little information was provided. Based on the classifications for the separate food categories, we classified schools as having students with healthier or unhealthier dietary intake during school hours, representing favorable and unfavorable outcomes respectively. Schools were only included in the analyses if we could classify the school for at least four out of the six categories for dietary intake. Therefore, we excluded one primary school from the QCA. Schools had a favorable outcome if at least four categories were classified as + or +−, and not more than one category as −. Schools received an unfavorable evaluation if these criteria were not met, and at least half of the categories (without missing values) were classified as −.

For secondary schools, we assessed three categories: (1) bread or snacks, (2) drinks (including all drinks, e.g., water and sugar-sweetened beverages), and (3) fruit. In secondary schools, it is also common to bring food from home. There is usually also a school canteen and food dispensers where students can purchase food and drinks, such as sandwiches, chips, and soda. Schools were included if information was available for at least two categories. We also included schools if they only had information available regarding bread/snacks, but only if the school staff indicated that this was healthy (+) or the opposite (–), so not +−. Secondary schools were classified based on the most frequent classification for the separate categories, i.e., a school that scored a + for bread/snacks and fruit but a − for drinks would be classified as having students with healthier dietary intake during school hours. We will refer to healthier and unhealthier behavior as the favorable and unfavorable outcomes, respectively. Three secondary schools were excluded from the QCA since two schools had very limited information regarding the dietary intake of students during school hours and one school could not be classified for the outcome. Therefore, we included fifteen primary schools and twelve secondary schools in our QCA. At the time of the school visits, the primary schools had, in total, 3271 students, and the secondary schools had 11,354 students.

#### 2.2.3. Conditions and Calibration

We determined the included conditions based on our research question, previous studies within the larger evaluation study [24,25], the target audience of SHP, existing literature, and the qualitative analysis of the interviews. The QCA should have a restricted number of conditions due to the exponential growth of possible combinations [26]. If the number of conditions increases but the sample size stays the same, there is a rise in the number of configurations of the conditions where the outcome is uncertain. It is usually recommended to include four to eight conditions if the sample size ranges from twelve to fifty cases [27].

For primary schools, we included the following conditions: whether a school implemented the *HS* program for nutrition, the degree of implementation of whole-school health promotion, the socioeconomic status of the parents of the students, parental support, and student support with regard to nutrition. For secondary schools, we included the same conditions, except for parental and student support. Instead, we included whether the school environment, i.e., the presence of food suppliers, was perceived as a hindering factor by the school staff. Indicators for the classifications are presented in Table 1. All conditions were coded as 0 or 1. A classification of 0 meant a low degree of implementation regarding nutrition, low SES, and low support among parents and students, whereas a value of 1 meant the opposite (high). For HS, 1 indicated that the school implemented the program for nutrition and 0 indicated that the school did not implement the program for nutrition.

#### 2.2.4. Data Analysis

We performed a QCA, using R version 4.3.1 [31] with the QCA package version 3.21 [32], to examine the relation between the favorable or unfavorable outcome and the possible configurations of conditions. First, we determined the ‘set memberships’ for the outcome and the conditions. We classified all conditions as 1 (fully in) or 0 (fully out), which is called a crisp set. Based on these set memberships, QCA is used to identify which (combinations of) conditions are necessary and sufficient for the outcome. QCA assumes asymmetry, i.e., if the presence of certain conditions leads to a favorable outcome; however, this does not necessarily indicate that the absence of the conditions leads to an unfavorable outcome [19]. Therefore, the favorable and unfavorable outcomes are examined separately.

Necessary indicates that when the outcome (i.e., Y) is present, the condition (i.e., X) is present as well (Y → X) [33]. Sufficient indicates that when a condition or a combination of several conditions is present, the outcome is also present (X → Y) [33].

##### Necessity

We identified single necessary conditions based on the scores for consistency, coverage, and Relevance of Necessity (RoN). These can all take values between 0 and 1, and the higher, the better. We used a threshold of 1 for consistency, as recommended for crisp sets [27,33]. Consistency indicates the proportion of schools with the outcome that have the specific configuration [34]. Therefore, a consistency score of 1 indicates that all schools with the outcome have the condition. Coverage and RoN are both indicators of trivialness. There are no strict thresholds, but we used 0.6 for coverage and 0.5 for RoN, which are usually used as the lower limits [26,34,35]. Coverage reflects whether the number of schools with the condition is higher than the number of schools with the outcome. Hence, coverage is calculated by dividing the schools with the specific configuration and the outcome by the total number of schools with the condition [34]. RoN is usually lower since it is more conservative than coverage because it also takes into account the difference in the number of schools with and without the condition [33].

##### Sufficiency

To examine sufficiency, i.e., which configurations of conditions lead to the outcome, we created a truth table for every outcome separately [Table A1, Table A2, Table A3 and Table A4 in Appendix A]. A truth table presents all possible configurations of conditions, such as for how many schools each configuration was observed and whether each configuration is sufficient for the outcome (i.e., leads to the outcome), is not sufficient, or is unknown. It also presents the consistency, which can range from 0 to 1. For the solution, truth table rows were included if consistency was ≥0.750, which is considered the lower limit for sufficiency [26,36]. For sufficiency, consistency is calculated by dividing the number of cases with the configuration and the outcome by the total number of cases with the configuration [34]. Next, we applied logical minimization to summarize the information derived from the selected truth table rows and construct the conservative, also called complex, solution for sufficiency. This means that to construct our solution, we did not include the truth table rows that were not observed in schools [33]. To illustrate logical minimization, we provide an example: if configurations ABC and AB~C (~is used to indicate ‘not’) both have outcome Y, then the classification of C is not of importance for the outcome. ‘And’ is usually depicted as ‘+’. Therefore, ABC + AB~C → Y can be reduced to AB → Y [33]. In case we identified a necessary condition, we compared the conservative solution to the intermediate solution, where we added a directional expectation for the necessary condition and the outcome [33,37].

For the final solution, we calculated the raw, unique, and solution coverage for the identified pathways for sufficiency, which can also range from 0 to 1. The higher the coverage, the higher the empirical relevance. Raw coverage shows to what extent the outcome is explained by a certain pathway [37]. The total solution coverage is the raw coverage for all pathways combined. Unique coverage is the solution coverage minus the raw coverage of the other pathways. The final step was to relate these findings back to the school data to enhance our comprehension of the results.

## 3. Results

### 3.1. Results QCA

We included fifteen primary schools and twelve secondary schools in our QCA. For primary education, we classified eight out of the fifteen schools as having students with healthier dietary intake during school hours; however, there was a clear division between the four schools with the healthiest dietary intake of students and the other four schools. For secondary schools, we classified seven out of the twelve secondary schools as having students with healthier dietary intake during school hours. The unobserved configurations of conditions, i.e., empty truth table rows, were 71.9% for primary schools and 62.5% for secondary schools. An overview of the truth table rows is presented in Table A1, Table A2, Table A3 and Table A4 in Appendix A.

#### 3.1.1. Primary Schools

For primary schools, we did not identify a necessary condition for healthier dietary intake of students during school hours. However, we found three sufficient pathways for the healthier dietary intake of students during school hours: (1) if all conditions were present or high (the implementation of the HS program for nutrition, the degree of implementation, SES, parental support, and student support), (2) if a school implemented the HS program for nutrition, but support among parents and students was absent and the SES was low, (3) if all conditions were absent, except for student support and a high SES.

For the unfavorable outcome, we identified one necessary condition, i.e., low support of parents (consistency = 1.000, coverage = 0.636, and RoN = 0.500). Additionally, we found three sufficient pathways for the unhealthier dietary intake of students during school hours: (1) if support among parents and students was low, and the school did not implement the HS program for nutrition and had a low degree of implementation, (2) if a school implemented the HS program for nutrition and had a high degree of implementation and high student support but SES was low and parental support was absent, (3) if a school implemented the HS program for nutrition, had high SES and student support, but a low degree of implementation and parental support was absent. For the unfavorable outcome, we compared the conservative to the intermediate solution because we identified a necessary condition; however, the results were the same.

#### 3.1.2. Secondary Schools

We did not identify any necessary conditions for secondary schools, but we found three sufficient pathways for secondary schools that led to the healthier dietary intake of students during school hours: (1) if a school implemented the HS program for nutrition but had low SES and had an impeding environment, (2) if a school did not implement the HS program for nutrition, had a low degree of implementation and an impeding environment, but had high SES, (3) if a school did not implement the HS program for nutrition but had a high degree of implementation, no impeding environment, and high SES. For the unfavorable outcome, we identified one pathway: (1) if a school did not implement the HS program for nutrition, had a low degree of implementation, a low SES, and an impeding environment.

### 3.2. Reflection on the QCA Results

#### 3.2.1. Primary Schools

An overview of the identified pathways is presented in Table 2, where we refer to primary school cases with a P, i.e., 1P. For the favorable outcome, the pathway with the highest coverage was achieved if all conditions were present or high (the implementation of the HS program for nutrition, the degree of implementation, SES, parental support, and student support). This was the case for four schools. All four schools reported some resistance when initially implementing a nutrition policy, but some school staff mentioned that it became easier over time when the policy existed for a couple of years. Additionally, these schools had personal contact with parents about the school’s nutrition policy, which appeared to be more effective in boosting engagement than organizing informational events. As an example, school 9P was one of the schools with this configuration, where bringing and consuming healthy food products appeared to be the social norm. They aimed to prevent resistance from parents by discussing with them that they implemented the HS program before their child enrolled in primary school. Additionally, the general SES of the school was high, but school staff noted that water is free, reflecting that someone’s financial situation does not necessarily have to be an issue for healthier dietary habits. The second pathway was if a school implemented the HS program for nutrition but support among parents and students was absent and the SES was low. This was the case for schools 4P and 15P. A reason why these schools still had rather good outcomes was because they provided free fruit multiple times per week. School 4P also provided free lunch at school on some days, but student support was low due to dissatisfaction with the provided meals. School 15P prohibited soda and cookies, and students, especially in lower grades, had to take these items home. Maintaining this rule became harder in the higher grades, particularly as older students enrolled from different schools and were therefore less inclined to adhere to the school’s nutrition policy. The last identified pathway was if all conditions were absent, except for student support and a high SES. School 12P was the only school with this pathway. School staff mentioned that they stimulated healthier dietary intake among the lower-grade students and, to a lesser extent, among the higher-grade students. However, there was no strict nutrition policy, but they believed this was not necessary since students did not consume a lot of unhealthy food.

There were also some schools that discussed the unhealthier dietary intake of students during school hours. For the unfavorable outcome, the highest coverage was observed for schools that did not implement the HS program, had a low degree of implementation, and had low support among parents and students.

School 10P illustrates this pathway, where school staff experienced a lot of resistance from parents. In general, both the parents and the students did not seem to care much about healthy nutrition. If the school adhered to a stricter nutrition policy, some parents would more strongly express discontent that their child was not allowed to eat restricted food items during school hours. The school staff noted that this consumed excessive time and negatively impacted teaching. Consequently, they opted to reduce their focus on students’ dietary intake. The second pathway was for schools that implemented the HS program for nutrition and had a high degree of implementation and high student support, but SES was low, and parental support was absent. This was observed for schools 2P and 7P. Both schools stimulated healthier dietary intake among students, but the school staff of both schools mentioned that if they did not continue to stimulate healthier nutrition, parents would no longer adhere to the school’s nutrition policy. The last identified pathway was if a school implemented the HS program for nutrition and had high SES and student support but a low degree of implementation and parental support was absent. This was only observed in school 8P. This school implemented the HS program and stimulated healthier dietary intake, for example, through the school guide; however, they believed it was mostly the responsibility of parents; therefore, they provided suggestions and free fruit and water bottles but did not have a strict nutrition policy. Teachers also experienced activities related to nutrition, such as stimulating water consumption during one week, as a heavy workload on top of many other obligations.

#### 3.2.2. Secondary Schools

An overview of the identified pathways is presented in Table 2, where we refer to secondary school cases with an S, i.e., 1S. In secondary schools, students generally appeared to have a less healthy dietary intake during school hours than students in primary school. Most schools reported that students consumed unhealthy products occasionally or more often. Additionally, fruit consumption was less frequently observed compared to primary schools, where most schools provided free fruit. Another difference was that, inside almost all secondary schools, we observed that it was possible to purchase unhealthy food products.

The pathway with the highest coverage for the healthier dietary intake of students during school hours was if a school implemented the HS program for nutrition but had low SES and an impeding environment. School 12S illustrates this pathway by implementing a policy regulating the portion size of unhealthy products, such as chips or soda. School staff supervised breaks to ensure compliance with the nutrition policy. Consequently, although students still consumed these products, their intake was restricted in quantity, signifying an improvement in dietary choices. The second pathway was if a school did not implement the HS program for nutrition, had a low degree of implementation and an impeding environment, but had high SES. This applied to two schools. One of these schools was school 9S, and the staff mentioned that the majority of students originated from higher SES backgrounds. As mentioned earlier, this factor frequently correlates with healthier dietary habits. This might explain why students had healthier dietary choices during school hours compared to other schools, despite the absence of certain conditions and the lack of implementation of the HS program for nutrition. The final pathway was if a school did not implement the HS program for nutrition but had a high degree of implementation, no impeding environment, and high SES, which applied to school 3S. School 3S did not implement the HS program for nutrition, but facilitated healthier food options and had additional water taps outside the toilet. The school also provided education with regard to nutrition. Their student council was also involved in decisions related to nutrition, such as healthier food options in the school canteen.

For the unfavorable outcome, we found one pathway: if a school did not implement the HS program for nutrition, had a low degree of implementation, a low SES, and an impeding environment. One of the schools with this configuration was school 5S. The school staff perceived nearby food providers and the parents as the most impeding factors, since they believed not all students were exposed to information about healthy nutrition and its importance within their household. School staff mentioned that they tried offering healthy products in their school canteen, but students would purchase unhealthy snacks at nearby food suppliers instead. Another school was school 11S, and an employee mentioned the following: “What strikes me, is that we talk about poverty (…), while children apparently have the resources to go to the supermarket and buy junk food”.

## 4. Discussion

The aim of our study was to examine under what conditions SHP, operationalized as implementing the HS program regarding nutrition, was related to healthier dietary intake during the school hours of primary and secondary school students.

For primary schools, we found that implementation of the HS program for nutrition was related to the healthier dietary intake of students during school hours in different contextual settings. Most schools that achieved a favorable outcome implemented the HS program for nutrition with a high degree of implementation in a favorable context, i.e., if support among students and parents was high, as well as the SES. The four schools that met these criteria were also the schools with students with the healthiest dietary intake during school hours. Nevertheless, it appeared that even in a more challenging school context, the HS program can still be positive for healthier dietary intake. This was observed in low SES schools, with low support among students and parents, regardless of the degree of implementation. We also observed healthier dietary intake if the HS program for nutrition was not implemented, but this was empirically less relevant since this was observed in only one school. This was the case for a high SES school, with high student support but a low degree of implementation and low support among parents.

Low support among parents was identified as necessary for less healthy dietary intake among students during school hours, emphasizing the important role of parents. Consequently, all schools where students had unhealthier dietary intake during school hours had low parental support, but other contextual factors could differ. The unfavorable outcome was most often observed in schools that did not implement the HS program, had low support among parents and students, and had a low degree of implementation, regardless of the school’s SES. However, the implementation of the HS program for nutrition resulted in an unfavorable outcome in some contextual settings. This was the case if these schools had a high degree of implementation and student support but low SES and parental support. Another combination where the implementation of the HS program for nutrition resulted in an unfavorable outcome was if high SES schools had student support but a low degree of implementation and no parental support.

For secondary schools, the favorable outcome was observed most often in schools that implemented the HS program and had a low SES and an impeding environment, regardless of the degree of implementation. This indicates that even if the degree of implementation is low and contextual factors are unfavorable, the implementation of the HS program can lead to a healthier dietary intake of students during school. Nevertheless, without implementing the HS program, it was still possible to obtain favorable results. This was the case for high SES schools, which had low implementation of SHP and an impeding school environment. One explanation could be that these students have a healthier diet in general due to their home environment [22]. In line with this finding, we found that if a high SES school has no impeding environment and a high degree of implementation, but did not implement the HS program, students also had a healthier dietary intake during school hours. This indicates that the high implementation of other whole-school health promotion initiatives, such as the Health Promoting Schools framework, might also be associated with the healthier dietary intake of students during school hours in high SES schools with no impeding environment.

On the contrary, schools that did not implement the HS program for nutrition and scored low on all other contextual factors (SES, environment, implementation) had students with unhealthier dietary intake during school hours. One of the school employees of a low SES school mentioned that despite a relatively high proportion of students coming from lower SES families, students still seem to have the financial resources to purchase unhealthy food products. This might indicate that not just monetary constraints within the household might play a role in the dietary behavior but maybe also other factors such as parental knowledge or attitude regarding healthy nutrition [38]. According to our results, if these schools would implement the HS program, even with a low degree of implementation, this would result in healthier dietary intake of students during school hours. Additionally, we did not identify any contextual settings for secondary schools where the implementation of the HS program for nutrition was related to the less healthy dietary intake of students during school hours.

It was remarkable that only low SES secondary schools were categorized as having an unfavorable outcome, but all schools with low SES that implemented the HS program had students with healthier dietary intake during school hours, even if other contextual conditions were unfavorable. Therefore, even though we want to emphasize that there was still room for improvement in all schools, we recommend the HS program to keep prioritizing low SES schools when assigning the additional support to implement the HS program, which is their current policy [20].

Additionally, the percentage of unobserved combinations of configurations was still high, indicating that we did not have information on the outcome for many possible configurations of conditions. This is common when performing a QCA [39,40,41]. For example, we did not include high SES schools that implemented the HS program regarding nutrition in our QCA for secondary schools. A very large N should be obtained to increase the number of conditions that can be included in the model. Nevertheless, increasing the number of schools does not automatically lead to a greater coverage of truth table rows, since some combinations of conditions are less probable to occur, and, therefore, are less likely to be observed [42]. Additionally, using a crisp set (where conditions are classified as either fully in (1) or fully out (0)) instead of a fuzzy set (partial membership is possible for the conditions, i.e., values between 0 and 1) simplifies the interpretation of the results and, therefore, can produce more straightforward results, which are more meaningful for policymakers [19]. However, the conditions, and therefore the results, contain less detailed information and, therefore, might be a less accurate description of the complex reality. For example, it is less suitable for identifying tipping points [43], which is an important characteristic of complex adaptive systems. The outcomes should also be dichotomized for QCA; however, we noticed a lot of variation within the groups, especially in primary schools. Therefore, subtle differences are difficult to grasp. This might have resulted in some pathways that seemed contradictory. For example, no student and parent involvement, together with a lower SES and the implementation of the HS program for nutrition led to a favorable outcome, while the same situation with student involvement and good implementation led to an unfavorable outcome. This might be a consequence of these forced classifications. It is also plausible that we did not include all relevant factors in our analyses. Nevertheless, the pathways with the highest coverage, indicating higher empirical relevance, were straightforward and were identified as most meaningful. For primary schools, it seems clear that the best results are obtained if all conditions are high or present; therefore, schools should strive to accomplish this. Additionally, in both primary and secondary schools, the implementation of the HS program for nutrition, irrespective of its degree of implementation, related to positive outcomes if all other conditions were minimal or absent. Therefore, even if these contextual factors are unfavorable, the HS program seems to be related to healthier dietary intake. This study also underlined the importance of parents’ and students’ home environment; therefore, we recommend schools to put additional effort into involving parents. Additionally, even though in some schools implementing the HS program seemed to be related to healthier dietary intake during school hours, we did not have any information on the dietary intake of students at home. Therefore, it is unknown whether the HS program might also impact the dietary intake of students outside school hours.

### Strengths and Limitations

Our study had some strengths and weaknesses. A strength of the QCA method is that it allows for the utilization of both qualitative and quantitative information. Consequently, we were able to combine data from multiple data collection methods, such as interviews and a questionnaire. Another advantage was data triangulation since we had multiple data sources to determine the set relations, i.e., the classification of the conditions. A limitation of the study was that some school staff had difficulties answering the question about the dietary intake of the students, and they did not always completely agree on whether the dietary intake of the students or certain food products could be considered healthy or unhealthy. We could also not classify all schools as having students with healthier dietary intake or not and were, therefore, not able to include all schools in our QCA. This resulted in information loss. Additionally, some questions remain because we did not observe all possible configurations of conditions for our outcome. For example, is the implementation of the HS program for nutrition related to healthier dietary intake of students during school hours in secondary schools with high SES in different contexts? We did not include high SES schools that implemented the HS program regarding nutrition in our QCA for secondary schools and could, therefore, not conclude about the relation between the HS program and dietary intake of students during school hours in these schools. This should be examined in further research, as well as the pathways in secondary vocational schools since the HS program is also implemented in these schools. We recommend to include a higher number of schools, to have a larger variety across conditions, and/or to include more relevant conditions. Another limitation of the study was that our results were on the school level and not on the individual level. We provide an overview of contexts in which the HS program is related to the healthier dietary intake of students in general. However, factors such as age, gender, and individual SES might moderate the association, resulting in differences between students. For the current study, we were unable to examine these associations on an individual level.

## 5. Conclusions

We conclude that implementing the HS program for nutrition appears to be related to the healthier dietary intake of students during school hours in most schools. For primary schools, the best results are obtained in high SES schools with high implementation, parental support, and student support, but the implementation of the HS program can also make a difference in low SES schools. Parental support was identified as an important condition; however, it is not necessary to obtain healthier dietary intake among students during school hours. These findings indicate that if secondary schools have unfavorable contextual factors, i.e., an impeding environment and low socioeconomic status, the implementation of the HS program for nutrition can result in a healthier dietary intake of students during school hours. Nevertheless, it remains challenging to examine the impact of SHP on the dietary intake of students during schools, while taking into account contextual factors.

## Figures and Tables

**Table 1:** Conditions for QCA and indicators for set membership.

Condition	Description and Rationale	Indicators for Set Membership
Healthy School	The implementation status of the HS program, specifically for the nutrition certificate. This condition is necessary to answer our research question.	1: If the school had the nutrition certificate in 2022, or there is an indication that the school implemented the HS program for nutrition based on the questionnaire and/or interview. 0: The school did not have the nutrition certificate in 2022, and there was no indication that the school implemented the HS program for nutrition based on the questionnaire and/or interview.
Implementation	The degree of the implementation of whole-school health promotion regarding activities regarding nutrition. The degree of implementation has been identified as an important factor in achieving the desired results in other studies.	1: If the degree of implementation is above average for the education sector, based on the original dataset [28], except if the nutrition score for adherence * is very low (<3). 0: If the degree of implementation is below average for the education sector, based on the original dataset, except if the nutrition score for adherence is very high (>5).
Socioeconomic status (SES)	The general SES of the parents. Previous studies within this project have identified SES as an important factor for dietary intake, as well as an extensive number of other studies.	Primary schools: 1: A low school weight [29] of 20–30 (if available), supported by the interview. 0: A high school weight of 30–40 (if available), supported by the interview. Secondary schools: 1: School disadvantage score [30] of 0 (if available, corrected for school size), supported by the interview. 0: School disadvantage score > 0 (if available, corrected for school size), supported by the interview
Parental support (only for primary schools)	Whether parents are actively and personally involved in the school’s activities regarding nutrition and provide mainly healthy food for their children during school hours has been identified in previous literature as an important factor and was frequently mentioned by the school staff as one of the most important factors.	High (1): Two criteria are met: (1) Parents are actively/personally involved in the school’s nutrition policy, e.g., parents are notified in person or through a personal note if they do not adhere to the guidelines. (2) Parents provide mainly healthy food/drinks for their children. Low (0): If the criteria for high are not met.
Student support (only for primary schools)	Whether students accept and support the school’s activities with regard to nutrition. The students are eventually the target group of the HS program.	High (1): If students address each other about their dietary intake and/or if students have positive responses to activities, or policy and environmental changes regarding nutrition. Low (0): If there is resistance among students or if negative reactions of multiple students are discussed.
Environment (only for secondary schools)	Whether the direct environment, e.g., the presence of food suppliers, of the school, was perceived as an important supporting or impeding factor in relation to the dietary intake of the students. The important role of food suppliers was discussed by most schools.	High (1): The school’s environment has not been mentioned as an impeding factor in relation to nutrition. Low (0): The school’s environment, such as the presence of supermarkets, has been mentioned as a hindering factor in relation to nutrition.

* Adherence is one of the seven dimensions measured using the questionnaire. Only the dimension of adherence produces scores for different health topics, one of them being nutrition. A higher score indicates a greater alignment of the implementation with the principles of the HS program.

**Table 2:** Solution for healthier/less healthy dietary intake during school hours of students in primary and secondary schools.

*Primary schools*											
**Conditions ¹**											
**HS**	**IMP**	**PAR**	**STU**	**SES**	**Outcome ²**	**Raw** **Coverage**	**Unique Coverage**	**Consistency**	**Solution Coverage**	**Solution Consistency**	**Cases**
1	1	1	1	1	1	0.500	0.500	1	0.875	1	3P,5P,6P,9P
1		0	0	0	1	0.250	0.250	1			4P;15P
0	0	0	1	1	1	0.125	0.125	1			12P
0	0	0	0		0	0.429	0.429	1	0.857	1	1P,10P;11P
1	1	0	1	0	0	0.286	0.286	1			2P,7P
1	0	0	1	1	0	0.143	0.143	1			8P
*Secondary schools*											
**Conditions ¹**											
**HS**	**IMP**	**SES**		**ENV**	**Outcome ²**	**Raw** **Coverage**	**Unique Coverage**	**Consistency**	**Solution Coverage**	**Solution Consistency**	**Cases**
1		0		0	1	0.429	0.429	1	0.857	1	1S;2S,12S
0	0	1		0	1	0.286	0.286	1			7S,9S
0	1	1		1	1	0.143	0.143	1			3S
0	0	0		0	0	0.600	-	1	0.600	1	5S,10S,11S

Note: HS = implements the HS program for nutrition, IMP = degree of implementation of school health promotion for nutrition, STU = support among students regarding nutrition; PAR = support among parents regarding nutrition, SES = socioeconomic status, ENV = environment. ^1^ 1 indicates high/present/favorable, 0 indicates low/absent/unfavorable. ^2^ 1 indicates healthier dietary intake during school hours; 0 indicates less healthy dietary intake during school hours.

## Data Availability

Data are contained within the article.

## Appendix A

**Table A1:** Truth table for primary schools for healthier dietary intake during school hours.

Healthy School	Implementation	SES	Parental Support	Student Support	Outcome *	N	Consistency	Cases **
1	1	1	1	1	1	4	1.000	3P,5P,6P,9P
1	0	0	0	0	1	1	1.000	4P
1	1	0	0	0	1	1	1.000	15P
0	0	1	0	1	1	1	1.000	12P
0	0	0	0	1	0	2	0.500	13P,14P
0	0	0	0	0	0	2	0.000	1P,10P
1	1	0	0	1	0	2	0.000	2P,7P
0	0	1	0	0	0	1	0.000	11P
1	0	1	0	1	0	1	0.000	8P

Note: 1 = high/favorable, 0 = low/unfavorable. N = number of cases with the configuration. * 1 = present, 0 = absent. ** Underlined cases are deviant, i.e., have the opposite outcome.

**Table A2:** Truth table for primary schools for unhealthier dietary intake during school hours.

Healthy School	Implementation	SES	Parental Support	Student Support	Outcome *	N	Consistency	Cases **
0	0	0	0	0	1	2	1.000	1P,10P
1	1	0	0	1	1	2	1.000	2P,7P
0	0	1	0	0	1	1	1.000	11P
1	0	1	0	1	1	1	1.000	8P
0	0	0	0	1	0	2	0.500	13P,14P
1	1	1	1	1	0	4	0.000	3P,5P,6P,9P
1	0	0	0	0	0	1	0.000	4P
1	1	0	0	0	0	1	0.000	15P
0	0	1	0	1	0	1	0.000	12P

Note: 1 = high/favorable, 0 = low/unfavorable. N = number of cases with the configuration. * 1 = present, 0 = absent. ** Underlined cases are deviant, i.e., have the opposite outcome.

**Table A3:** Truth table for secondary school for healthier dietary intake during school hours.

Healthy School	Implementation	SES	Environment	Outcome *	N	Consistency	Cases **
1	0	0	0	1	2	1.000	2S,12S
0	0	1	0	1	2	1.000	7S,9S
1	1	0	0	1	1	1.000	1S
0	1	1	1	1	1	1.000	3S
1	1	0	1	0	3	0.333	4S,6S,8S
0	0	0	0	0	3	0.000	5S,10S,11S

Note: 1 = high/favorable, 0 = low/unfavorable. N = number of cases with the configuration. * 1 = present, 0 = absent. ** Underlined cases are deviant, i.e., have the opposite outcome.

**Table A4:** Truth table for secondary school for unhealthier dietary intake during school hours.

Healthy School	Implementation	SES	Environment	Outcome *	N	Consistency	Cases **
0	0	0	0	1	3	1.000	5S,10S,11S
1	1	0	1	0	3	0.667	4S,6S,8S
1	0	0	0	0	2	0.000	2S,12S
0	0	1	0	0	2	0.000	7S,9S
1	1	0	0	0	1	0.000	1S
0	1	1	1	0	1	0.000	3S

Note: 1 = high/favorable, 0 = low/unfavorable. N = number of cases with the configuration. * 1 = present, 0 = absent. ** Underlined cases are deviant, i.e., have the opposite outcome.
